# Circulating Tumor DNA in Gastric Adenocarcinoma: Future Clinical Applications and Perspectives

**DOI:** 10.3390/ijms24119421

**Published:** 2023-05-29

**Authors:** Giulia Grizzi, Massimiliano Salati, Maria Bonomi, Margherita Ratti, Lauren Holladay, Maria Caterina De Grandis, Daniele Spada, Gian Luca Baiocchi, Michele Ghidini

**Affiliations:** 1 Oncology Unit, ASST Cremona, 26100 Cremona, Italy; giulia.grizzi@asst-cremona.it (G.G.); maria.bonomi@asst-cremona.it (M.B.); mratti.cremona@gmail.com (M.R.); daniele.spada@asst-cremona.it (D.S.); 2 Department of Oncology and Hematology, University Hospital of Modena, 41124 Modena, Italy; maxsalati@live.it; 3 Anne Burnett Marion School of Medicine, Texas Christian University, Fort Worth, TX 76129, USA; laurenholladaymd@gmail.com; 4 Oncology Unit 1, Veneto Institute of Oncology IOV—IRCCS, 35128 Padua, Italy; mariacaterina.degrandis@iov.veneto.it; 5 Department of Surgery, ASST Cremona, 26100 Cremona, Italy; gianluca.baiocchi@unibs.it; 6 Oncology Unit, Fondazione IRCCS Ca’ Granda Ospedale Maggiore Policlinico, 20122 Milan, Italy

**Keywords:** gastric cancer, liquid biopsy, circulating tumor DNA, minimal residual disease, MRD, ctDNA

## Abstract

Gastric cancer (GC) is still one of the most aggressive cancers with a few targetable alterations and a dismal prognosis. A liquid biopsy allows for identifying and analyzing the DNA released from tumor cells into the bloodstream. Compared to tissue-based biopsy, liquid biopsy is less invasive, requires fewer samples, and can be repeated over time in order to longitudinally monitor tumor burden and molecular changes. Circulating tumor DNA (ctDNA) has been recognized to have a prognostic role in all the disease stages of GC. The aim of this article is to review the current and future applications of ctDNA in gastric adenocarcinoma, in particular, with respect to early diagnosis, the detection of minimal residual disease (MRD) following curative surgery, and in the advanced disease setting for treatment decision choice and therapeutic monitoring. Although liquid biopsies have shown potentiality, pre-analytical and analytical steps must be standardized and validated to ensure the reproducibility and standardization of the procedures and data analysis methods. Further research is needed to allow the use of liquid biopsy in everyday clinical practice.

## 1. Introduction

Gastric cancer (GC) is the sixth most common type of cancer and ranks third concerning mortality rate worldwide [1]. In 2020, more than 1 million new cases and 768,793 deaths were estimated globally [2]. In the next years, these burden estimates will increase due to the aging population and the growth of high-risk groups. Surgery and systemic chemotherapy remain the cornerstones of treatment, in addition to target therapy, immunotherapy, and radiotherapy in selected patients [3].

Currently, the only biomarkers with clinical applications for treatment decisions are HER2 overexpression and amplification, PDL1 combined positive score expression (CPS), and microsatellite instability-high status (MSI-H) [4,5]. In the advanced setting, the ToGA trial, published in 2010, demonstrated an overall survival (OS) benefit when trastuzumab was added to first-line chemotherapy in HER 2-positive gastric or gastro-esophageal junction (GEJ) cancer [6]. After nearly a decade of negative results, nivolumab was the first PD-1 inhibitor to show superior OS in combination with chemotherapy (versus chemotherapy alone) in previously untreated patients with advanced gastro-esophageal adenocarcinoma (GEA) [7].

In early and localized settings, MSI-H/mismatch repair deficiency (dMMR) was associated with a better prognosis and apparent reduction in benefit from perioperative or adjuvant chemotherapy [8]. MSI-H/dMMR status is a strong predictive factor of benefit from immunotherapy regardless of the stage and the line of treatment [9]. Recently, Zolbetuximab, an antibody targeting claudin-18.2, combined with mFOLFOX6, statistically significantly prolonged progression-free survival (PFS) and OS in patients with CLDN18.2 positive/HER 2 negative, locally unresectable or metastatic GEA [10]. Advances in molecular classification have helped to characterize the biology of different subtypes of GC. In 2014, the Cancer Genome Atlas (TCGA) research network proposed four molecularly different GC subtypes: Epstein–Barr virus-positive (EBV+), microsatellite instable (MSI), genomically stable (GS), and chromosomal unstable (CIN) [11]. Nevertheless, their application to everyday clinical practice is still limited, with the exception of the predictive and prognostic role of EBV and MSI subtypes [12].

Although, in recent years, evidence from clinical trials supporting the use of target therapy and immunotherapy in neoadjuvant, adjuvant, and advanced settings for GC has rapidly expanded, five years survival is still dismal with a high rate of late diagnosis, systemic relapses after surgery, and primary or secondary resistance to treatment. Therefore, new techniques to improve early diagnosis and real-time monitoring of disease are required. Liquid biopsy is the sampling and analysis of non-solid biological tissues including blood, urine, saliva, ascites, stool, and pleural and cerebrospinal fluids [13,14,15]. The fundamentals of liquid biopsy are that the tumor will release into the bloodstream or other fluids cell-free nucleic acids (DNA or RNA) and other fragments such as circulating tumor cells (CTCs), circulating tumor microemboli (CTM), exosomes, proteins, cytokines or tumor-educated platelets (TEPs) [13]. These sources contain molecular information that can be used to diagnose cancer, define prognosis, quantify tumor burden, and identify targetable alterations to predict sensibility or resistance to treatments [14,15,16]. When compared to traditional tissue biopsy, liquid biopsy is less invasive, requires fewer samples, and can be repeated over time, which allows for longitudinal monitoring of tumor burden and molecular changes [17].

In this review, we summarize the evidence of the role of circulating tumor DNA (ctDNA) in gastric adenocarcinoma early diagnosis, detection, and monitoring of minimal residual disease (MRD) after radical treatment and in advanced disease.

## 2. Characteristics of ctDNA and Detection Methods

Plasma DNA is constituted of DNA fragments bound to proteins to prevent degradation. In healthy patients, the main source of cell-free DNA (cfDNA) is hematopoietic cells, while in cancer patients, a variable percentage of circulating DNA derives from cancer cells (i.e., ct-DNA). The main source of plasma DNA is cell death (i.e., apoptosis or necrosis); however, other biological processes may contribute such as active release from living tumor cells [18]. Ct-DNA constitutes <1% of cfDNA, is generally smaller than non-tumoral plasma DNA (143-145 base pair (bp) versus 166 bp), and has a half-life of fewer than 2 h. Its clearance takes place primarily in the liver, although the kidney may clear smaller fragments [19]. As ctDNA can be released by different tumor subclones, it is more representative of the molecular heterogeneity of a given cancer than a solid biopsy performed on a single site. Two strategies have emerged for studying ctDNA:

(1) A targeted approach that identifies specific mutations previously detected in the primary tumor. It is fast, highly specific, and can detect very low allele frequencies. It can be used for early disease detection, monitoring of early-stage tumors, or MRD analysis. The difficulty of this approach is that it requires abundant information about the tumor genome [17,18,19,20];
(2) An untargeted approach that implies a wide genome or exome sequencing and does not require a prior characterization of the primary tumor. This approach is more useful in advanced or metastatic settings in order to identify actionable molecular alterations or mutations related to therapeutic resistance. The disadvantage is that high concentrations of ctDNA are required with an overall low sensitivity [17,18,19,20]. Implemented methodologies include NGS, digital PCR, quantitative PCR (qPCR), and mass spectrometry [17]. As ctDNA levels are influenced by multiple tumor- and host-related variables, choosing the appropriate ct-DNA assay and its timing to address a specific clinical or scientific issue is essential. At present, no assay can be considered fit for all purposes. The CfDNA fraction fluctuates according to tumor stage, type, and site. In the case of higher tumor burden and/or extra-cranial progression, solid tumors will more likely shed ct-DNA into the bloodstream if compared to tumors with limited burden and or intracranial progression only [17]. Moreover, detectable ct-DNA levels have been found in >75% of patients with advanced pancreatic, bladder, ovarian colorectal, breast, melanoma, hepatocellular, head and neck, and gastroesophageal cancers, but in less than 50% of patients with brain, renal, thyroid, and prostate cancers [21].

The involvement of the peritoneal membrane is a frequent condition in GC and is correlated with a worse prognosis. Ascites is available in large quantity, can be extracted from the patient using minimally invasive procedures, and the detected DNA may originate from necrotic tumor cells. A few retrospective data suggested that ascitic fluid contains a larger amount of tumor-derived DNA compared to plasma ctDNA. Additionally, Wu et al. found that ascites supernatants had a higher actionable mutation rate and more actionable alterations than the plasma of 26 patients affected by gastrointestinal cancers with paired samples [22].

Surgery, radiotherapy, oncologic treatments, or inflammation may also influence ct-DNA concentrations. Collection, storage, and processing of the samples should always be performed following validated standard operating procedures (SOPs) to avoid DNA degradation and minimize variability in the assay results [17,19]. False-negative results (either due to low DNA concentration or low sensitivity of a chosen assay or poor shredding from the primary tumor) should always be taken into account when interpreting the results of the assay. Equally, false positive results that may be caused by CHIP (clonal hematopoiesis of indeterminate potential) should be ruled out, especially when testing tumor suppressor genes such as DNA repair genes [20]. Recommendations to implement liquid biopsy clinical use and to develop reliable diagnostic, prognostic, and predictive tools have been published [17,23].

## 3. Clinical Applications of ctDNA in Gastric Cancer

### 3.1. Early Diagnosis

Diagnosing cancer at its earliest stage when still localized to the site of origin and amenable to a curative-intent approach most likely represents the most transformative clinical application for liquid biopsy [24]. In fact, in a non-invasive and increasingly cost-effective manner, blood-based ctDNA analysis would offer the opportunity to anticipate cancer diagnosis before stage IV, contributing to a considerable reduction in cancer-related deaths [25]. This is particularly relevant in GC as outside high-risk Asian countries, where cost-effective screening programs have led to a substantial decrease in cancer-specific mortality, no screening modalities are currently recommended for GC, resulting in late diagnosis and poor outcome in two-thirds of cases [5]. Moreover, the low sensitivity and specificity of conventional serum tumor markers, including CEA and Ca-19.9, prevent their use as screening tools for GC [26]. The quantitation and sequencing of circulating cell-free DNA (cfDNA) with the identification of genetic and epigenetic alterations are the most extensively studied biomarkers for early cancer detection so far.

With regard to the absolute circulating DNA concentration, mean levels of plasma cfDNA have been reported to be significantly higher in GC than in healthy individuals (106.88 ± 12.40 ng/mL vs. 79.78 ± 8.12 ng/mL, *p* < 0.001), and among patients, it has been shown to correlate with higher tumor burden (i.e., tumor size, T stage, TNM stage) [27,28,29]. Interestingly, the level of serum cfDNA has been shown to be nearly 6 times more elevated in stage I GC compared to healthy controls and to proportionally increase with the disease stage [30]. Consistently, Lan and colleagues identified an optimal cut-off value of 2700 copies/mL, achieving a sensitivity of 68.9% for cfDNA (*p* < 0.001), in a cohort of 429 patients with GC and 95 healthy controls [31]. Concerning the potential diagnostic utility of tumor-specific alterations, the detection of copy number variants (e.g., HER2 amplifications) may also be important in the screening for GC, at least for the molecularly defined subset of HER2-positive GC. A study by Grenda et al. indeed reported that the HER2 gene copy number measured using the qPCR method was significantly higher in the sera of patients than in healthy people (*p* = 0.01) and that blood-based HER2 testing distinguished between these groups with a sensitivity and a specificity of 58% and 98%, respectively. Of interest, it has been shown that cfDNA levels in GC are more elevated than those in benign and pre-cancerous conditions, such as gastric adenomas. Based on this, a study incorporating cfDNA is underway in South Korea (NCT04665687) to identify biomarkers discriminating between neoplastic and non-neoplastic gastric lesions [30,31,32,33].

Changes in DNA methylation are early events in carcinogenesis that may even anticipate the occurrence of genetic mutations and are highly cancer-specific. Aberrant DNA hypermethylation of CpG islands is an epigenetic alteration known to occur in GC patients. Thus, methylation-based sequencing has gained traction as a tool for early GC diagnosis following the promise to be superior to genomic mutations for cancer detection [34,35,36,37,38]. While initial reports showed promising results for single methylation biomarkers in GC, a relatively higher detection efficiency was achieved with multiple ones. To this end, a study by Ren et al. identified 153 cfDNA methylation biomarkers, including KCNQ5, CABIN1, and DOCK10, for detecting GC using a genome-scale DNA methylation analysis method (MCTA-Seq) on the plasma samples from patients with GC (*n* = 89) and controls (n = 82), as well as 28 pairs of GC and adjacent non-neoplastic tissues. In this study, MCTA-Seq detected GC at an early stage with a sensitivity of 40%–60% (stage I–III) and specificity of 92% [39]. Similarly, following a whole-methylome discovery on case-control tissues, another study developed a plasma-based, three-marker panel (ZNF569, C13orf18, ELMO1) able to detect GC in 86% of cases with 95% specificity [40]. Thanks to its capability to detect initial signals for multiple cancers from cfDNA, a novel approach called multi-cancer early detection is undergoing extensive investigation for the early diagnosis of a variety of cancers. Specific to gastrointestinal cancers (GI), EpiPanGI Dx is a pan-GI diagnostic assay based on differentially methylated regions, which showed robust diagnostic accuracy when predicting the tissue of origin for six different GI tumors. The panel, developed from DNA methylation data on tissue and validated in plasma-derived cfDNA, displayed a prediction accuracy of 0.85–0.95 for most GI cancers, including GC (AUC 0.90) [41].

Other interesting data came from the “Galleri blood test”, which is a multicancer early detection test developed by GRAIL Inc. (Menlo Park, CA, USA). Within the Circulating Cell-free Genome Atlas study (CCGA; NCT02889978), this tumor-agnostic blood test was validated by detecting cancer signals across more than 50 tumor types, with a specificity of 99.5% and an overall accuracy of cancer signal origin prediction in true positives of 88.7% [42]. Regarding GC, the overall sensitivity was 66.7% (20/30), but it dropped to 16.7% and 50% in stage I and II, respectively, and was lower compared to stage I liver/bile duct (100%) cancers and pancreatic cancer (61.9%).

Despite recent technological advances, the exploitation of ctDNA analysis for early detection of GC lags behind other malignancies mainly due to its suboptimal sensitivity. More data from larger ongoing studies such as the PATHFINDER trial (NCT04241796) are needed to ascertain whether ctDNA may complement conventional diagnostics to screen for GC. As the field is rapidly evolving, there is hope that novel multi-parametric tests may at least in part overcome the limitations of currently available tools.

### 3.2. Minimal Residual Disease

The evaluation of MRD in GC is an opportunity for lowering recurrence rates in the resected disease. Since the advent of blood-based circulating tumor DNA (ctDNA), different efforts have been made in order to evaluate its use in GC [43]. Currently, there are two approaches for MRD assessment: a tumor-informed one that starts from the genomic sequencing of the primary tumor and a tumor-agnostic one that is uninformed by the mutations in the primary tumor [44]. Although more expensive than tumor-naïve tests, tumor-informed approaches are preferable as far as they are able to determine MRD with both high sensitivity and specificity, with the possible identification of tumor-specific variants with a very low variant allele frequency of 0.01%. Differently, tumor-agnostic tests are less sensitive and able to detect tumor-specific variants for a VAF between 0.1 and 1%. On the whole, sequencing of multiple tumor regions increases the sensitivity of the test and the possibility to detect ctDNA variants [44]. However, the discrepancy between the primary tissue and ctDNA profile may be caused by cancer heterogeneity. Indeed, in order to precisely characterize the clonal composition of tumors, the VAF must be converted to the cancer cell fraction (CCF), that is, the fraction of cancer cells within which the variant is present. Events appearing in all cancer cells with a CCF of 100% are considered clonal, while in the case of CCF < 100%, events are subclonal [45]. Therefore, the predominance of a specific clone masking the minority clones with lower CCF together with the arbitrariness of biopsies in the primary tissue may contribute to a significant discrepancy between the primary tissue and ctDNA profile [46]. Moreover, MRD cannot be immediately detected in plasma after surgery, and the evaluation of serial timepoints of plasma after resection allowed the identification of ctDNA variants with a progressive increase in VAF in relapsed patients. Usually, ctDNA detection typically precedes radiological relapse by a median time of 3–8 months [44].

Among studies of ctDNA detection for MRD monitoring in resected GC, a prospective cohort of 46 Chinese patients with stage I–III resected GC was evaluated using tissue and longitudinal plasma samples. CtDNA was detected in 45% of treatment-naïve plasma samples and was correlated both with clinicopathological features and postoperative DFS and OS. The preoperative T stage was associated with preoperative ctDNA positivity (*p* = 0.006), with all patients showing ctDNA in the postoperative follow-up who eventually relapsed (100% relapse for ctDNA positivity versus 32% for ctDNA-negative, *p* = 0.0015). Evidence of ctDNA positivity at any time during follow-up (median follow-up 29.1 months) was associated with worse DFS and OS (HR = 14.78, 95% CI, 7.991–61.29, *p* < 0.0001 and HR = 7.664, 95% CI, 2.916–21.06, *p* = 0.002, respectively). The sensitivity and specificity of immediate postoperative ctDNA positivity were 39 and 100% for the prediction of recurrence, respectively. MRD identification preceded radiographic relapse of disease by a median of six months. Moreover, all patients with detectable ctDNA but equivocal radiological imaging eventually had a recurrence, with a suggested role for MRD evaluation in the case of unclear results of imaging during follow-up [47].

Differently, Leal et al. evaluated a subgroup of 50 Western patients enrolled in the phase III randomized CRITICS trial on perioperative chemotherapy versus preoperative chemotherapy with postoperative chemoradiotherapy. All patients received 3 cycles of neoadjuvant chemotherapy, and a tumor-agnostic approach with the evaluation of 58 genes was used in order to identify tumor-specific alterations in the circulation with parallel deep sequencing of cfDNA and white blood cells (WBCs). Subsequently, ctDNA alterations were identified, and hematopoietic changes detected in WBCs were deleted. After the exclusion of mutations from clonal hematopoiesis of indeterminate potential (CHIP), preoperative ctDNA positivity was a significant predictor of recurrence (HR, 3.0; *p* = 0.012) as well as shorter OS (HR, 2.7; *p* = 0.03). Similarly, after CHIP mutation filtering, postoperative ctDNA positivity was associated with a significantly higher risk for recurrence (HR, 21.8; *p* < 0.001). Therefore, after the distinction of ctDNA from other cfDNA alterations, ctDNA harbored a predictive role in clinical outcomes of perioperative chemotherapy and cancer resection [48]. Results from smaller studies are reported in Table 1.

Some studies on the topic are ongoing. A prospective Asian study is recruiting 100 patients receiving radical gastrectomy, with ctDNA and other tumor markers being collected within 14 days before surgery and then tested after gastrectomy in the scheduled interval. The primary outcome of the study is the evaluation of the sensitivity and specificity of MRD detection using ctDNA as a biomarker (NCT05029869). Differently, a single-arm pilot study aimed at evaluating adjuvant pembrolizumab plus trastuzumab in HER2+ esophagogastric tumors with Persistent ctDNA following curative resection. CtDNA was tested at least four weeks after completion of the surgery and standard perioperative and/or adjuvant therapy. To be considered eligible for trastuzumab/pembrolizumab or trastuzumab therapy, the patients must have had positive ctDNA in a time-lapse of 4 weeks-8 months after completion of the appropriate standard of care therapy. The primary endpoint of the study was the proportion of patients with ctDNA clearance at 6 months during trastuzumab/pembrolizumab. The study was terminated early due to a lack of accrual (NCT04510285).

On the whole, ctDNA evaluation after GC resection is promising for early prediction of recurrence. However, few prospective and large studies have been conducted so far and consistent data is missing; therefore, justification for routine clinical use is a challenge at present.

### 3.3. Advanced Disease

CtDNA detection and monitoring are useful to provide different types of information in advanced GC without the need for invasive procedures such as tissue biopsy. First of all, ctDNA can be used to detect predictive biomarkers for the treatment response as, for example, HER2 overexpression/amplification, PDL1, and FRGFR2 overexpression. Moreover, quantification of the ctDNA level is also useful for identifying the mechanisms of resistance to the ongoing therapy and predicting the response of the disease before radiological examinations. The use of ctDNA allows for finding specific targetable genomic alterations so that it is possible to offer personalized treatment to every single patient. However, up to now, the ctDNA-based approach has not been part of routine practice to direct physician treatment decisions. Quantitatively dynamic changes in ctDNA levels can be used for monitoring disease evolution, and they are also a prognostic biomarker in advanced GC. In a large study including more than 1500 patients with metastatic GC and collected samples of ctDNA, those with a >50% decline in the max variant allelic frequencies (maxVAF) after the beginning of the treatment had a better mOS compared to patients with <50% decline (13.7 months vs. 8.6 months, *p* = 0.02, HR 0.3 95% CI 0.1–0.8) [53]. The same evidence was confirmed also for those treated with immunotherapy: patients with less than the median maxVAF of 3.5% showed better mOS. Moreover, patients with HER2 amplification demonstrated improved OS when treated with anti-HER2-specific drugs. In another study, plasma samples from 30 patients were collected and analyzed with whole-genome sequencing. Overall, 77% had detectable ctDNA, and it was found that a higher ctDNA concentration was correlated with a shorter OS [54]. These data support the clinical utility of ctDNA as a prognostic biomarker for advanced GC.

In order to identify predictive biomarkers for a response, the use of ctDNA is a current area of meaningful research for several tumor types, including GC. Two studies showed different results with respect to the association between the detection of chromosomal instability using ctDNA and response to chemotherapy. The first one evidenced that a reduction in the copy number instability score correlates with a higher response, as calculated using ctDNA after treatments. In addition, the response was deeper in those with chromosomal instability (ORR 59%) when compared to patients with chromosomal stability (ORR 32%) [55]. However, another study found no correlation between chromosomal instability and response to chemotherapy, which is why further and prospective trials are required to confirm this hypothesis [54]. HER2 overexpression and/or amplification are mandatorily tested in advanced GC in order to activate a targeted anti-HER2 therapy with trastuzumab and with the antibody–drug conjugate trastuzumab–deruxtecan (T-DXd) [6,56]. However, the intra-tumoral heterogeneity in HER2 expression on tissue biopsy may represent a significant problem, and the use of a plasma-based method of detection could be a strategy to overcome this issue and identify a more complete molecular profile for each tumor. Two studies assessed the concordance between ctDNA and tissue-based HER2 amplification, showing significantly different results of 91.2% [57] and 61% [53], respectively. In HER2-positive GC, ctDNA can be useful for predicting and monitoring the response of the disease to specific anti-HER2 drugs. In a prospective trial of 24 HER2-positive metastatic GC treated with trastuzumab, ctDNA showed that changes in HER2 copy number were correlated with tumor response prior to imaging examinations [58]. In the phase 2 DESTINY-gastric 01 trial, patients with a baseline ctDNA ERBB2 copy number above 6.0 showed a T-DXd great response (ORR 75.8%), while patients below 6.0 revealed a lower activity of the drug (ORR 40.8%) [59]. Larger and prospective studies are needed to validate the predictive role of ctDNA HER2 amplification and the optimal copy number cut-off. In HER2-positive mGC, ctDNA is also useful for identifying mechanisms of resistance to anti-HER2 therapies. In 15 patients, an NGS-ctDNA detection was performed to investigate the mechanism of resistance after the progression of the first-line, trastuzumab-based systemic treatment [53]. The analysis found a loss of HER2 amplification in 73% of patients, while the appearance of possible resistance mutations such as KRAS, PIK3CA, and BRAF-alterations was described in those with persistence of HER2 amplification. In another study of 17 patients who progressed during trastuzumab treatment, the amplification of MET and NF1 mutations was identified as a possible mechanism of resistance, even with the persistence of HER2 amplification [58]. Prior to and during an anti-HER2 treatment, the monitoring of HER2 expression using ctDNA was a promising biomarker for identifying early progressions and overcoming the use of invasive tissue-based procedures. Since the combination of chemo-immunotherapy became the new first-line standard of care in PDL1-positive, HER2-negative advanced GC [7], a marked interest was shown in the possibility of identifying and monitoring the PDL1 status in a non-invasive way. In a study including 46 patients who underwent an anti-PD1 inhibitor, the decrease in ctDNA higher than 25% in maxVAF showed longer mPFS (7.3 months vs. 3.6 months) and higher ORR (53% vs. 13%) [60]. Patients with baseline ctDNA mutations in TGFBR2, RHOA, and PREX2 and treated with immunotherapy revealed worse mPFS when compared to those with a wild-type phenotype (*p* < 0.05). Moreover, patients with mutations in FGFR4, MET, CEBPA, and KMT2B showed a superior incidence of immune-related adverse events. In a study of 61 patients with mGC treated with pembrolizumab, a serial ctDNA analysis was collected, and a reduction in ctDNA level at 6 weeks was found to be predictive of therapy benefit and PFS [61]. Two important biomarkers are known to be predictive of the response to immunotherapy: EBV+ and MSI [53,62]. The dynamic changes in EBV-DNA detected using ctDNA were assessed in a study of 2760 advanced GC in an Asian population. In tissue-based, EBV-associated GC, only 52% of them had a detectable EBV-DNA using ctDNA. Moreover, it was found that EBV-DNA levels correlated with a radiological response. In conclusion, the measurements of ctDNA-based EBV-DNA were not useful for identifying EBV-associated GC because of the low concordance between plasma and tissue analysis, but they may be of interest as a predictor for response in patients with known GC associated with EBV [63]. A few data are available about the possibility to detect MSI using liquid biopsy. Two different studies investigated the correspondence between microsatellite instability detected with ctDNA in patients known to be MSI-High using IHC and tissue-based NGS. In the first study, ctDNA-NGS identified six/six (100%) patients known to be MSI-high by directly sequencing microsatellite regions [53]. In the second one, the highly sensitive ddPCR was able to detect MSI in the cfDNA of two/five patients with T3/T4 GEA, while the other three/five patients, known to be MSI-H on tissue-based samples, had smaller T1/T2 tumor mass [64]. Despite the limited evidence, this represents a promising tool when it comes to using liquid biopsy for identifying this genomic alteration, which is relatively infrequent but highly targetable. FGFR2 overexpression is a molecular target that can be found in 5–10% of advanced GC [65]. Recently, the phase 2 FIGHT trial showed greater mPFS (9.5 months vs. 7.4 months) in FGFR2-positive mGC treated with the anti-FGFR2 bemarituzumab plus FOLFOX compared to FOLFOX alone [66]. In this study, 16% of FGFR2 overexpression was detected using ctDNA, thus suggesting that the use of liquid biopsy to identify this molecular target may be correlated with response to anti-FGFR drugs. Further data are needed to validate this evidence, and the phase 3 trial (FORTITUDE-101 NCT05052801) is ongoing.

**Figure 1:**
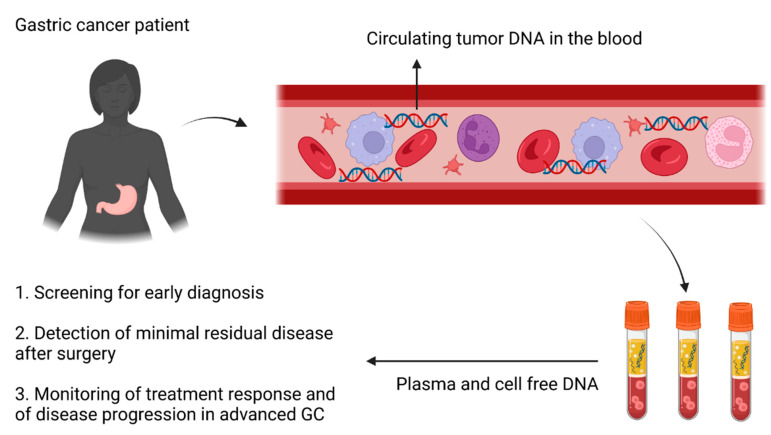
Clinical applications of liquid biopsy for GC. The release of circulating tumor DNA into the bloodstream can be used for: 1. early diagnosis; 2. detection of minimal residual disease after surgery; and 3. monitoring treatment response and disease progression in advanced GC.

Even if there are limited therapeutic targets in GC, the use of ctDNA is functional to detect genomic alterations that could guide the choice of personalized treatments. In the umbrella VICTORY trial, tissue samples were analyzed in order to detect 10 possible targetable alterations and allocate patients to specific interventional studies. Moreover, ctDNA was collected and analyzed at baseline and longitudinally during treatments [67]. Regarding MET amplification, the study revealed a concordance between tissue and ctDNA of nearly 90%. Furthermore, patients treated with the anti-MET inhibitor savolitinib showed a reduction in ctDNA levels before radiological evidence of the disease response. Additionally, the copy number of MET amplification on ctDNA was more predictive for PFS in patients treated with savolitinib compared to the same tissue-based information. The use of ctDNA is a promising field in advanced GC to overcome the problem of tumor heterogeneity and to detect predictive biomarkers for response and/or genomic alterations with the aim that every single patient may have a personalized treatment. Additionally, the monitoring of ctDNA levels could predict the response to the disease earlier than radiological examinations. Despite the aforementioned data, all these hypotheses need further evidence, which has to be derived from larger and prospective studies. Figure 1 summarizes the clinical applications of liquid biopsy for GC.

## 4. Main Challenges and Future Perspectives

Despite liquid biopsies that provide valuable information about GC, numerous issues must be solved for their reliable clinical application.

One of the main challenges in the development of liquid biopsies for GC is the low sensitivity and specificity of this test [15]. GC is a heterogeneous tumor, and the number of CTCs and ctDNA in the blood has high variability and distribution among different patients. Another limitation is the low specificity of ctDNA due to the high background noise from non-tumor-derived biomarkers in body fluids, such as the identification of cfDNA derived from normal tissues, which can lead to false-positive or false-negative results [68]. Furthermore, using NGS methods such as TAm-Seq, Safe-SeqS, and CAPP-Seq enables the detection of multiple rare mutations in ctDNA simultaneously and may improve sensitivity but, at the same time, may lead to false positives and altered genetic information [69]. Improving pre-analytical steps can reduce the background of wild-type DNA and may produce more reliable results [70]. Moreover, researchers are exploring the use of multiple biomarkers combined with machine learning algorithms to improve the accuracy of liquid biopsies for GC [71]. Lacking standardized protocols for sample collection, processing, and analysis represents a major obstacle to the clinical promotion of liquid biopsies for GC and can affect the quantity and quality of these biomarkers [72]. For example, the time from blood draw to plasma separation can affect the concentration of ctDNA in the plasma. Moreover, the detection and quantification of biomarkers are strongly influenced by the detection method, platform, and data analysis. The absence of clear guidelines for liquid biopsies prevents standardization of this technique, making results incomparable. Therefore, standardization of pre-analytical and analytical procedures is necessary to ensure the reproducibility and comparability of liquid biopsy results [73]. It is also unclear whether liquid biopsies provide a reliable representation of the entire tumor and its heterogeneity, including all genomic clones within an individual tumor or a specific sub-region of the tumor. Liquid biopsy can capture only a small fraction of tumor cells or biomarkers, which may not represent the entire tumor heterogeneity. Therefore, multiple liquid biopsies over time may be required to capture the dynamic changes in tumor composition and monitor the disease progression [74]. The analysis of liquid biopsies markers combined with modern imaging techniques and already existing biomarkers might be helpful in the near future for early cancer identification and individualized cancer treatment [75]. Another major concern about the use of liquid biopsies for GC is that current liquid biopsy trials included a small number of patients and had a short validation time. A large-scale clinical trial validation is mandatory [76]. Considering all these critical issues, new biomarkers have been investigated to replace or integrate liquid biopsies. Currently, exosomes, non-coding-RNAs, and tumor-educated platelets represent the new trends in the field of liquid biopsies [15]. Exosomes are small (30–140 nm) extracellular vesicles secreted by almost all types of cells and released into the extracellular environment through processes of fusion. They are now recognized as a fundamental way for cellular communication by transmitting proteins, DNA, mRNA, and non-coding RNAs [77]. It is believed that they have a main role in gastric cancer development and progression. Since exosomes are detected in various biofluids, such as blood, saliva, urine, and peritoneal fluid, they seem to be a great platform for the development of liquid biopsies. Noncoding RNAs, such as microRNAs (miRNAs), long noncoding RNAs (lncRNAs), and circular RNAs (circRNAs), can be packaged into exosomes, which allows them to remain stable and be found in the extracellular environment, vehiculating messages between cells and tissues [77]. In the last few years, many clinical trials tested exosomes and exosomal RNAs as diagnostic biomarkers for GC [78,79,80]. Generally, in these studies, blood samples were obtained from GC patients and healthy people, and quantitative reverse transcription PCR was used to measure the level of exosomal RNAs [78,79,80]. The optimal candidate was represented by those exosomal RNAs with significant expression differences between patients with GC and healthy controls. Those exosomal RNAs with calculated favorable values for sensitivity, specificity, and area under the ROC curve were reported as potential diagnostic biomarkers [78,79,80,81]. To improve the efficacy of these biomarkers as a diagnostic tool, a combination of miRNA is often used in clinical trials.

The prognostic role of exosomal RNAs is also under investigation in clinical trials [82]. The correlation between their expression and survival represents a major topic, but only a few studies have identified the mechanism behind the correlation. For example, the strict relationship between exosomal PD-L1 and worse OS in GC patients was found in the suppressive effects induced by exosomal PD-L1 on the immune status of GC patients [83].

Tumor-educated platelets (TEPs) represent one of the last discovered liquid biopsy components. TEPs are modified by tumors in multiple ways and act to help cancer cells grow and protect against metastatic spreading [84]. During the interaction with tumor cells, platelets become “educated “, resulting in altered RNA. In 2015, pan-cancer research on TEPs was performed by Best et al., developing a distinct once-signature for six primary cancer types, including colorectal cancer and hepatobiliary cancer [85]. Significant differences in terms of RNA profiles were noticed between TEPs and normal controls for multiple tumor types with high accuracy in cancer diagnosis [85]. To now, no application of this marker in GC has been found, but it is still under investigation. Figure 1 summarizes the different clinical applications of liquid biopsy for GC.

## 5. Conclusions

Compared to the limitations of conventional tissue biopsies, liquid biopsies can provide valuable information about the genetic and molecular characteristics of gastric cancer, tumor heterogeneity, genomic alteration, and tumor progression [15]. Liquid biopsies have several advantages over traditional tissue biopsy, including their ability to detect cancer at early stages and monitor the disease in real time. They have emerged as a promising non-invasive alternative to traditional diagnostic methods, allowing for most personalized treatment approaches [68]. As described in our review, the main applications of liquid biopsies in gastric cancer are represented by the prediction of prognosis, the evaluation of treatment efficacy, the achievement of an early diagnosis, the selection of patients suitable for target therapies, and, more recently, for immunotherapy [14,80].

Although liquid biopsies have shown potentiality before their incorporation into clinical practice, pre-analytical and analytical steps must be standardized and validated to ensure reproducibility and standardization of the procedures and data analysis methods [14,15,68,72,80]. Moreover, the low sensitivity and specificity of this technique prevent a larger application in gastric cancer. Prospective clinical trials designed with uniformity in procedures, including a larger number of patients with homogeneous cases, are mandatory to reveal the actual role of liquid biopsies and their application in gastric cancer settings [14,15,68,80].

## Figures and Tables

**Table 1:** Studies evaluating circulating tumor DNA and minimal residual disease in resected GC.

Author, Year	Number of pts	Study Design	Results
Kim, 2019 [49]	25	WGS → Tumor tissue qPCR → ctDNA testing in serum (before surgery, at months 1, 3, 6, 9, and 12 after surgery)	- Median lead time ctDNA + vs. imaging relapse: 4.05 months. - ctDNA + associated with relapse in 12-month postop period (*p* = 0.0294).
Ko, 2021 [50]	49	qPCR → ctDNA evaluation for *LINE1* fragments, ligation-mediated PCR → methylation proportion of *LINE1* postop	High postop concentration of *LINE1* associated with: - Shorter RFS (*p* = 0.009); - Shorter OS (*p* = 0.04).
Shoda, 2017 [51]	4	RT-PCR and dd-PCR → Postop levels of *HER2* + *RPPH1* (control gene) copy number	Four pts relapsed, all with *HER2*-to-*RPPH1* ratio above the cutoff.
Ling, 2013 [52]	72	RT-PCR → *XAF1* hypermethylation (matched tumor-ctDNA)	Overall, 12 pts relapsed, 10 pts with negative to positive *XAF1* methylation status on ctDNA.

ctDNA: circulating tumor DNA; postop: postoperative; dd-PCR: digital droplet PCR; pts: patients; qPCR: quantitative polymerase chain reaction; RFS: relapse-free survival; RT-PCR: real-time polymerase chain reaction; OS: overall survival; WGS: whole genome sequencing.

## Data Availability

No new data were created or analyzed in this study. Data sharing is not applicable to this article.
